# Enhancing clinical documentation with ambient artificial intelligence: a quality improvement survey assessing clinician perspectives on work burden, burnout, and job satisfaction

**DOI:** 10.1093/jamiaopen/ooaf013

**Published:** 2025-02-21

**Authors:** Michael Albrecht, Denton Shanks, Tina Shah, Taina Hudson, Jeffrey Thompson, Tanya Filardi, Kelli Wright, Gregory A Ator, Timothy Ryan Smith

**Affiliations:** Department of Internal Medicine, University of Kansas Medical Center (KUMC), Kansas City, KS 66160, United States; Department of Family Medicine and Community Health, University of Kansas Medical Center (KUMC), Kansas City, KS 66160, United States; Abridge, Philadelphia, PA 19102, United States; Department of Internal Medicine, University of Kansas Medical Center (KUMC), Kansas City, KS 66160, United States; Department of Biostatistics and Data Science, University of Kansas Medical Center (KUMC), Kansas City, KS 66160, United States; Department of Neurosurgery, University of Kansas Medical Center (KUMC), Kansas City, KS 66160, United States; Information Technology Services, University of Kansas Health System, Kansas City, KS 66160, United States; Department of Otolaryngology and Head and Neck Surgery, University of Kansas Medical Center (KUMC), Kansas City, KS 66160, United States; Department of Pediatrics, University of Kansas Medical Center (KUMC), Kansas City, KS 66160, United States

**Keywords:** artificial intelligence, ambient intelligence, documentation, professional burnout, electronic health records

## Abstract

**Objective:**

This study evaluates the impact of an ambient artificial intelligence (AI) documentation platform on clinicians’ perceptions of documentation workflow.

**Materials and Methods:**

An anonymous pre- and non-anonymous post-implementation survey evaluated ambulatory clinician perceptions on impact of Abridge, an ambient AI documentation platform. Outcomes included clinical documentation burden, work after-hours, clinician burnout, and work satisfaction. Data were analyzed using descriptive statistics and proportional odds logistic regression to compare changes for concordant questions across pre- and post-surveys. Covariate analysis examined effect of specialty type and duration of AI tool usage.

**Results:**

Survey response rates were 51.9% (93/181) pre-implementation and 74.4% (99/133) post-implementation. Clinician perception of ease of documentation workflow (OR = 6.91, 95% CI: 3.90-12.56, *P* <.001) and in completing notes associated with usage of the AI tool (OR = 4.95, 95% CI: 2.87-8.69, *P *<.001) was significantly improved. Most respondents agreed that the AI tool decreased documentation burden, decreased the time spent documenting outside clinical hours, reduced burnout risk, and increased job satisfaction, with 48% agreeing that an additional patient could be seen if needed. Clinician specialty type and number of days using the AI tool did not significantly affect survey responses.

**Discussion:**

Clinician experience and efficiency was improved with use of Abridge across a breadth of specialties.

**Conclusion:**

An ambient AI documentation platform had tremendous impact on improving clinician experience within a short time frame. Future studies should utilize validated instruments for clinician efficiency and burnout and compare impact across AI platforms.

## Background and significance

Electronic health record (EHR) implementation has led to unintended consequences, including increased documentation burden, burnout,^1^^,^^2^ and increased stress among clinicians.^3^ Studies indicate that 70% of healthcare providers experience stress related to EHR use, with inadequate time for documentation nearly tripling the odds of burnout.^4^ Ambulatory physicians spend 27% of their clinical day in direct contact with patients, dedicating nearly twice that time to EHR and administrative responsibilities, with chart review, documentation, and order entry ranking as the topmost activities.^5^^,^^6^ While documentation time varies among individual clinicians within each specialty, clinicians in medical specialties and primary care spend on average at least twice as much time on notes as those in surgical specialties.^6^ This imbalance contributes to an additional 1-2 hours of administrative work outside the workday,^5^ adversely affecting work-life balance and increasing burnout risk.^2^ There is a pressing need to explore innovative solutions that can alleviate the documentation burden on healthcare providers. Traditional documentation methods—manual typing, templated text, copy/paste, speech recognition, and human scribes—offer varying degrees of efficiency. Speech recognition and transcription methods have been associated with reduced documentation time compared to manual entry techniques.^7^ In addition, a randomized controlled trial in a family practice setting found that in-person human scribes improve time spent charting, perceived chart quality and chart accuracy, and time to note closure.^8^ Human scribes may be helpful for the documentation experience but may be cost-prohibitive for many practices.^9^ Use of these tools may unintendedly add time to a clinician’s workday. For example, speech recognition and manual typing are typically performed after a clinic visit has concluded.

Ambient AI documentation platforms are emerging technologies that utilize automatic speech recognition and generative AI to summarize clinical conversations into structured notes.^10^ These platforms promise to alleviate documentation burden by seamlessly integrating into the clinical workflow without requiring additional clinician effort during or after patient encounters.

## Objective

This quality improvement study aims to evaluate the impact of an ambient AI documentation platform, Abridge, on clinicians’ perceptions of documentation burden, after-hours work, burnout risk, and job satisfaction across various specialties. By assessing these factors, we seek to determine whether ambient AI can serve as an effective solution to the documentation challenges faced by clinicians.^10^

## Materials and methods

### Study design and participants

We conducted an analysis of survey data administered to physicians and advanced practice practitioners (clinicians) at the University of Kansas Medical Center (KUMC). Clinicians’ perceptions of documentation workflow, risk for burnout, job satisfaction, and timely completion of notes before and after use of the ambient AI documentation tool, Abridge, were assessed. 181 of 1255 credentialed medical staff members were enrolled. Members of physician leadership as well as physician informatics and ambulatory practice committees were prioritized for access to the tool, with word- of- mouth driving subsequent provisioning. Clinicians from 30 medical specialties were included in the study. For analysis, these specialties were grouped into 3 categories: primary care, medical subspecialty, and surgical subspecialty. Clinicians who received access to the tool were encouraged but not mandated to use the AI platform for documentation. These clinicians were still allowed to use other documentation tools available to them, such as speech-to-text technology, other ambient AI technology, or manual typing. The licenses to use the Abridge software were purchased by the health system and provided at no cost to clinicians.

### Ethical approval and consent

The KUMC Institutional Review Board approved the study as a quality improvement project, exempting it from full review. All participants provided informed consent electronically before completing the surveys. Patient consent for recording was obtained per institutional policy, which includes written consent for all technology used during clinical encounters. In Kansas and Missouri, the states that KUMC provides the majority of its patient care, it is not required for the patient to consent for audio recording of their visit.

### Ambient AI platform description

Abridge is an ambient AI platform that summarizes medical conversations for clinicians and patients across multiple care settings. KUMC was one of the first organizations in the country to pilot the use of Abridge in clinical practice. Our institution also has helped provide feedback to the vendor, helping them improve their ambient AI platform over time.

During the study, clinicians used an Abridge smartphone application to record patient-clinician conversations. When Abridge was first implemented at our institution, an Abridge iPhone app was available, but not an Android app. For the small number of users at our institution who used Android devices, iPhone devices were provided by our institution. Toward the latter part of the study period, an Abridge Android app did become available, and Android users could use their own devices from that point forward. There were no limitations in the ability to use the Abridge app based on iPhone or Android operating system versions during the study.

**Figure 1.**
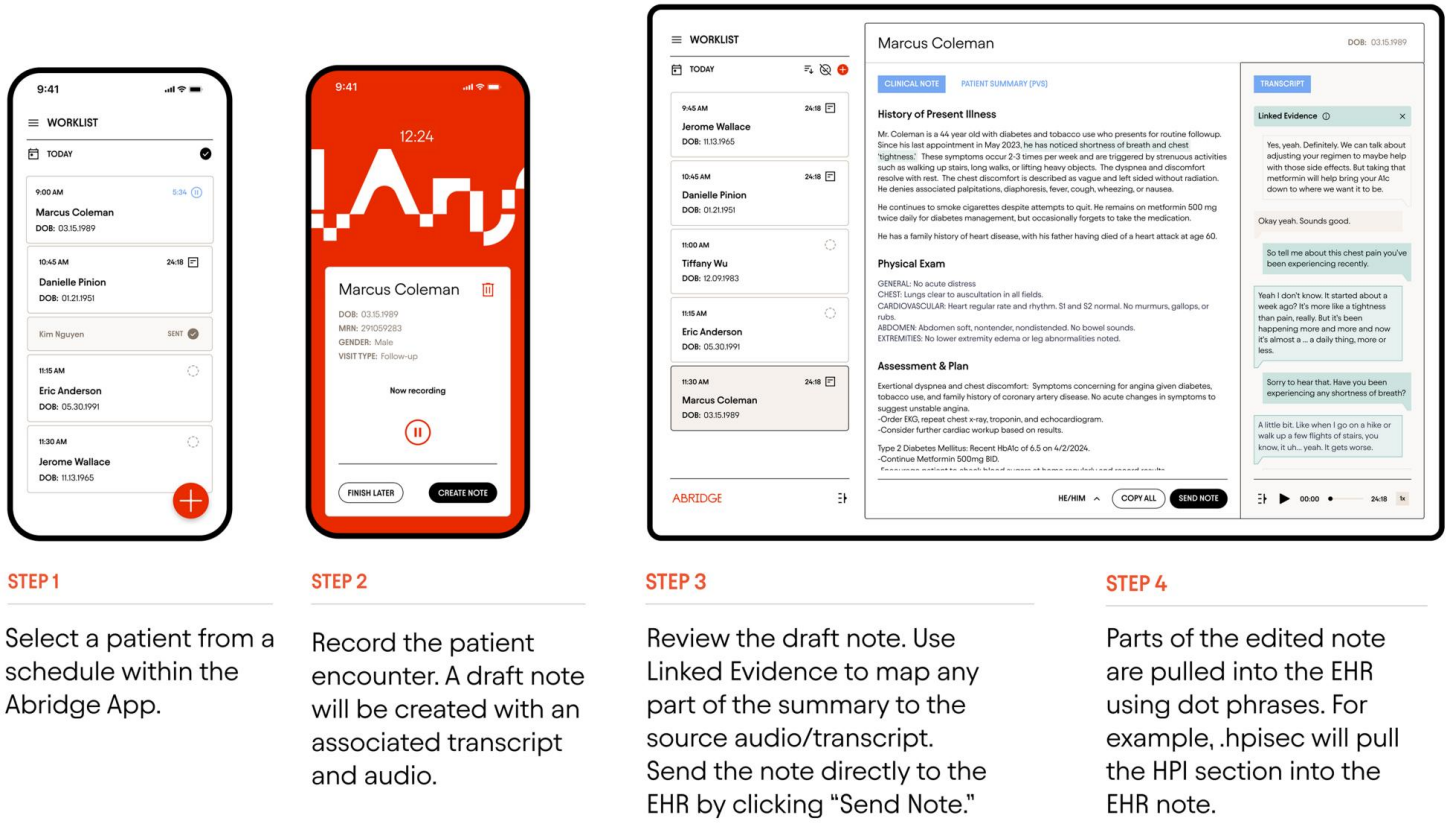
Sample workflow using Abridge platform. While full integration from Epic mobile app (Haiku) directly to notes in Epic is available, depicted here is the partially integrated workflow which was used during the study period. Figure courtesy of Abridge.

The use of Abridge to draft a clinical note is straightforward. Clinicians use the Abridge mobile application to select a patient from their EHR (Epic)-integrated clinic schedule. The decision whether to use Abridge for a particular patient visit is left to the discretion of the clinician. The clinician starts recording with the ability to pause and toggle between different patient encounters as needed. Clinicians then use a web editor to view and edit the AI-drafted note. Evidence that led to the summary can also be called up in a single click that brings up related sections in the transcript and audio to allow for real-time verification and trust in the summary. Once viewing and any editing necessary is complete, dot phrases are used to pull the draft note directly into the clinician's note template in the EHR. Figure 1 visually demonstrates the Abridge workflow.

### Survey instrument

The survey questions were developed by 2 physician informaticists and a business analyst for quality improvement and operations considerations. Survey questions were developed organically by the project team, although informed by literature that concerned provider burnout and documentation burden. The pre-implementation 8-item survey was administered to each clinician just prior to their first use of the AI documentation platform. The overall intent of this survey was to determine the general experience of note documentation of each provider before using the new tool. The initial usage date of the technology and, consequently, the date for administering the pre-implementation survey, varied among participants, ranging from April 2023 to February 2024. Another 11-item survey was developed with the intent of examining the clinician experience post-implementation of the ambient AI tool. The purpose of this survey was to assess clinician perception of benefits of the tool, as well as whether use of the tool should be expanded to a larger number of clinicians across the institution. The questions between the pre- and post-implementation surveys were not the same, although several questions were similar. The complete pre- and post-intervention surveys as well as complete survey item response distributions are provided in the online Appendix S1.

The post-implementation survey was distributed to all clinicians who had completed at least 5 clinical encounter recordings using Abridge. This survey was emailed to clinicians on February 27, 2024, with a follow-up prompt sent on March 1, 2024, and the survey closed on March 2, 2024. Clinicians had been using the AI platform for a median of 92 days prior to taking the post-implementation survey (IQR: 66 to 172 days, min = 16 days, max = 334 days). Clinical documentation workflow tool utilization was assessed via a multiple mark survey item. A 5-point Likert scale ranging from “Strongly Disagree” to “Strongly Agree” was used for survey items that pertained to documentation experience. There was an additional option of “Not relevant to my experience.” Participants that selected this option for 1 or more questions was excluded from analysis (*n* = 1 for pre-intervention survey and *n* = 2 for post-intervention survey). Each participant completed all questions for the surveys. The pre-intervention survey was anonymous and, therefore, the total and percentages of each specialty type depict those providers who could have taken the survey, not necessarily those who took the survey. Since the post-intervention survey was not anonymous, the number and percentages of each specialty type depict the values for those who took the survey. Furthermore, not everyone who completed the pre-implementation survey conducted at least 5 clinical encounters by the time the post-survey was administered. Therefore, there was a fewer number of participants who were offered the post-implementation survey than the pre-implementation survey.

### Statistical analysis

To compare the 2 matched pre- and post-survey questions, we used proportional odds logistic regression (POLR). POLR was also used to look at the effect of specialty type and number of days of use of the digital scribe on post-survey responses. We calculated odds ratios (ORs) and 95% confidence intervals (CIs) from the model coefficients. Statistical significance was set at a *P*-value of <.05. Descriptive statistics were also calculated. All statistical analyses were performed in R version 4.3.2^11^ within RStudio.

## Results

Of the initial 181 clinicians who were offered access to the ambient AI documentation platform, 93 completed the pre-implementation survey (51.9% response rate). The post-implementation survey was completed by 99 of the 133 clinicians who were offered the survey (74.4% response rate). Table 1 depicts the demographics of those participants who completed the post-intervention survey. Demographics for participants who completed the pre-intervention survey are not available, as the pre-intervention survey was anonymous. Since the 133 individuals who were sent the post-implementation survey were considered “adopters” of the AI tool (because they had completed at least 5 clinical encounters using Abridge), the level of adoption of the AI tool at our institution was 133/181 (73.5%).

**Table 1.** Demographics of post-intervention survey participants (*N* = 99).

**Age in years median (IQR)** ^a^	41 (37-46)
**Years in practice median (IQR)**	12 (8-17)
**Years at institution median (IQR)**	4 (2-8)
**Sex *N* (%)**	
Male	48 (48%)
Female	51 (52%)
**Practitioner type *N* (%)**	
Physician	86 (87%)
Advanced Practice Provider	13 (13%)
**Specialty type *N* (%)**	
Primary care	34 (34%)
Medical subspecialty	41 (41%)
Surgical subspecialty	24 (24%)
**Total**	99 (100%)

a Interquartile range

**Figure 2.**
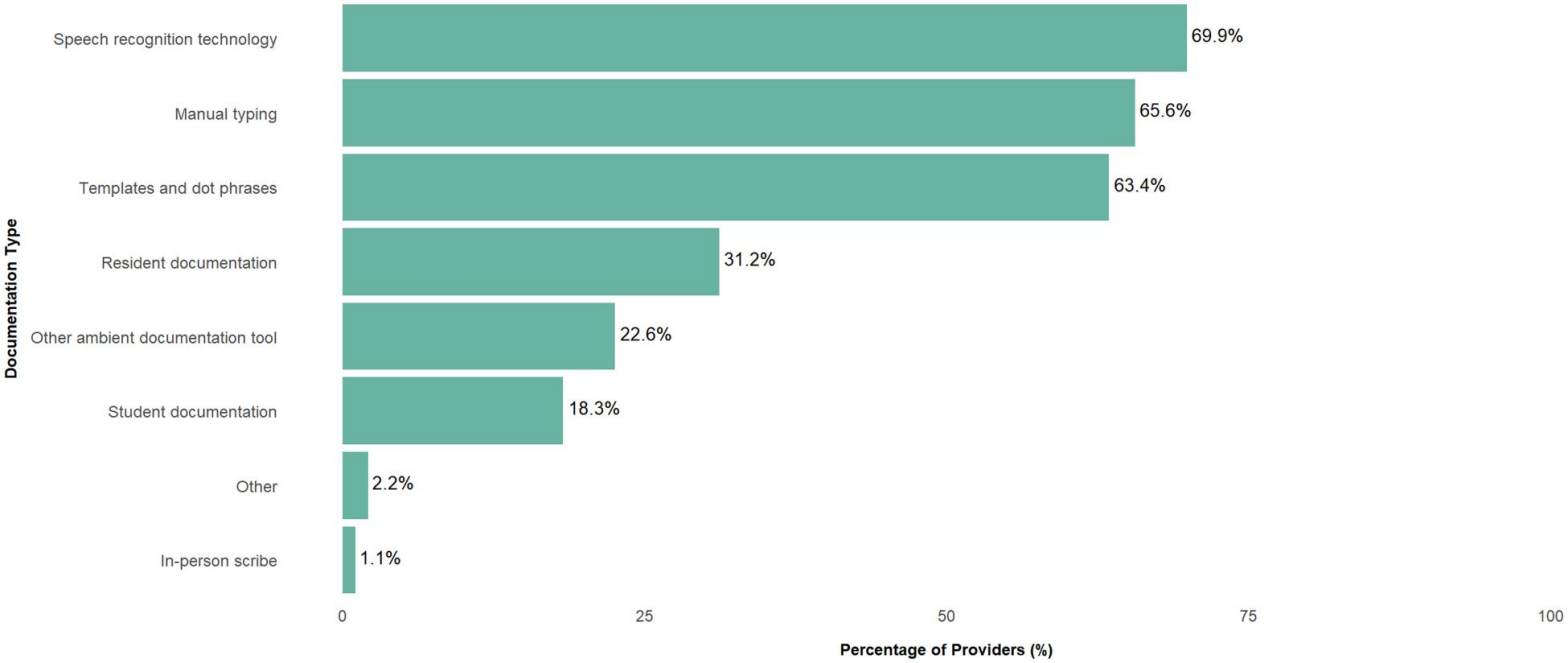
Percentage of clinicians utilizing various documentation workflow tools at baseline.

Before using the AI platform, 77/93 (82.8%) of respondents agreed or strongly agreed that they regularly spent time documenting outside of clinical hours and 70/93 (75.2%) agreed or strongly agreed that they were at risk for burnout due to documentation. In addition, at baseline over 60% of respondents cited speech recognition, manual typing, and templates and dot phrases as the predominant tools used for clinical documentation (Figure 2).

Concordant questions between pre- and post-implementation surveys were analyzed collectively to assess the impact of the ambient AI tool on clinical workflow. There was a 6.91 times higher odds of clinicians reporting a higher level of agreement that they find the documentation workflow easy in the post-intervention group compared to the pre-intervention group (CI: 3.90-12.56 *P *<.001). There was a 4.95 times increased odds of clinicians reporting a higher level of agreement with the statement that they could complete the note before the next patient visit (CI: 2.87-8.69, *P *<.001). Table 2 displays a comparison of the concordant questions between the pre- and post-implementation surveys.

**Table 2.** Proportional odds logistic regression analysis comparing concordant pre- and post-intervention survey items.

Pre-intervention survey item	Post-intervention survey item	**Odds ratio (95% CI** ^a^ **)**	*P*-value
I find my current documentation workflow easy to use.	Abridge has made my current documentation workflow easy to use for patient visits.	6.91 (3.90-12.56)	<.001
I usually complete the note before the next patient visit.	With Abridge, I could complete the note before the next patient visit.	4.95 (2.87-8.69)	<.001

a CI = confidence interval.

**Figure 3.**
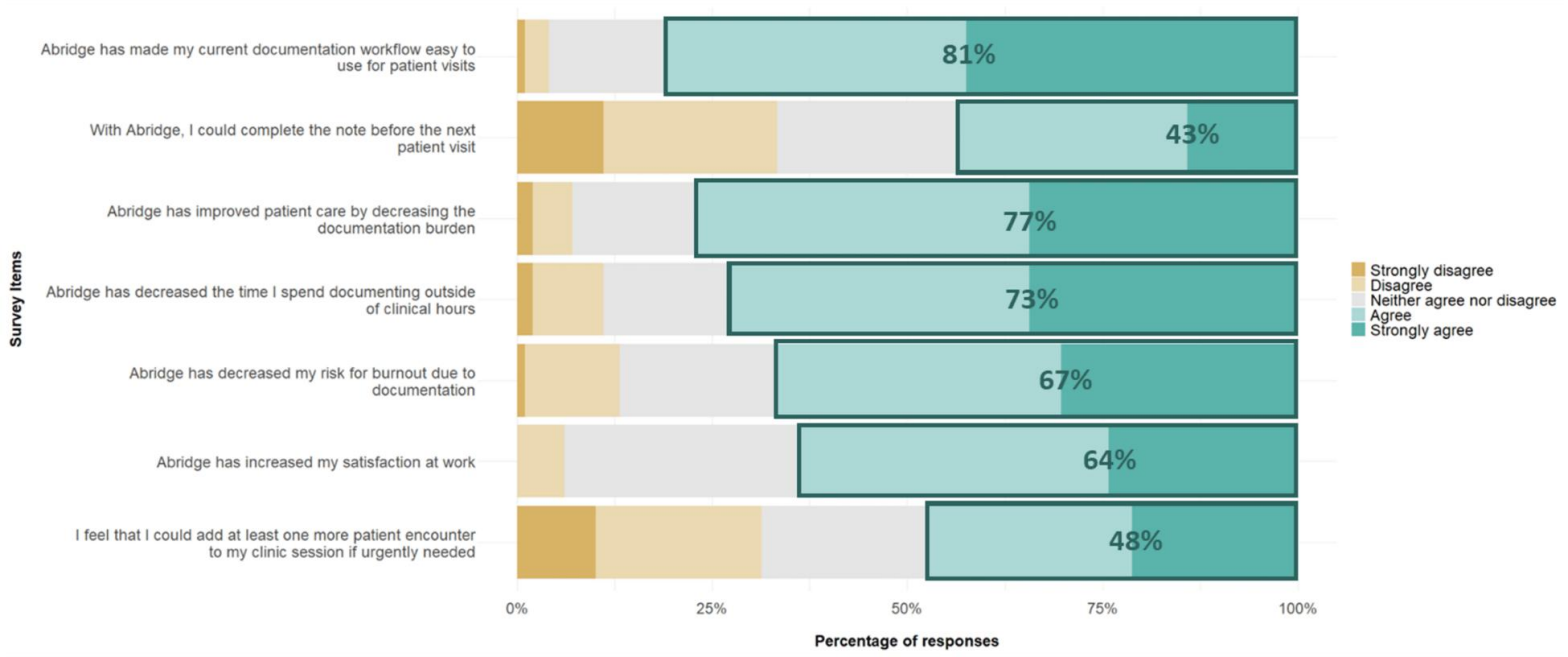
Post-intervention survey response percentages.

Figure 3 displays a descriptive summary of responses from the post-survey. In the post-implementation survey, respondents agreed or strongly agreed 80/99 (81%) of the time that the ambient AI platform had made their current documentation workflow easy to use, enabled them to complete the note prior to the next visit (43/99, 43%), improved their perception of patient care through decreased documentation burden (76/99, 77%), decreased their time spent documenting outside clinical hours (72/99, 73%), reduced their risk for burnout due to documentation (66/99, 67%), and increased their satisfaction at work (63/99, 64%). 47/99 (47%) of respondents agreed or strongly agreed that at least one more patient encounter could be added to clinic sessions if urgently needed. POLR analysis on post-implementation survey questions revealed that post-implementation survey responses did not differ significantly by specialty type (primary care, medical subspecialty, or surgical subspecialty) or number of days of use of the AI platform.

## Discussion

To our knowledge, this is the first multi-specialty study evaluating clinician perspectives on use of a fully ambient AI platform for clinical documentation. Use of the platform was associated with 7 times greater odds of clinicians reporting a higher level of agreement that their documentation workflow is easy and 5 times greater odds of clinicians reporting a higher level of agreement that they can complete documentation before the next patient encounter. In addition, most clinicians surveyed agreed that using the AI platform increased satisfaction at work, decreased documentation burden, decreased time spent documenting outside of clinical hours, and decreased risk for burnout. There was no evidence of a difference across responses when stratified by specialty type or length of use of the AI tool. Furthermore, the positive effects in this study were noted despite approximately 70% of providers already using speech recognition technology at our institution at baseline. Overall, these findings reflect that providers at our institution who used ambient documentation perceived it to be beneficial for their documentation workflows. While our study was approved as a quality improvement study and not necessarily considered generalizable outside our institution, the findings provide evidence that ambient AI technology not only reduces documentation burden and risk of burnout among primary care clinicians, but also benefits medical and surgical subspecialists.

There are several reasons why the ambient AI tool could have been perceived by clinicians to be beneficial in our study. First, the lack of multitasking (as may be required when documenting or using speech recognition while with the patient) and reduced need to recall encounter details by memory may have allowed clinicians to provide more dedicated attention to their patients. Providers may have sensed a greater amount of face-to-face engagement with patients, as ambient AI platforms allow for less interaction with a computer during ambulatory encounters. Second, features inherent to the tool itself such as deep integration of ambient AI into the EHR-based workflow could have also contributed to the noted improvements in workflow ease and note completion efficiency. Third, the reduction in after-hours documentation time attributed to ambient AI usage may have alleviated the strain on clinicians’ work-life balance, potentially reducing burnout risk. The perceived increase in job satisfaction could stem from the sense of support and assistance provided by AI tools in the often-demanding environment of clinical practice. Overall, the multifaceted benefits reported by clinicians highlight the potential of ambient AI documentation platforms to address longstanding challenges in healthcare documentation and improve clinician well-being.

The survey results on the impact of ambient AI documentation tools reveal a divide among providers regarding their ability to see more patients during a clinic session. Approximately half of the respondents felt confident they could add at least one more patient without concern. Protection of perceived wellbeing and control of workload may have disincentivized some respondents from affirming the ability to add patients to their schedules. We underscore that the primary goal of implementing such technology should be to alleviate provider burnout and improve work-life balance, rather than solely to increase patient volume. It should also be noted that without a similar baseline pre-implementation survey question, it is not possible from our study to differentiate whether clinicians answered affirmatively that they could add another patient to the clinic schedule was due to the AI tool intervention or some other factor.

There have been few peer-reviewed published studies to date on the effects of ambient AI documentation technology in clinical practice, and they were generally limited to a specific specialty. An observational study of 110 primary care providers using the ambient AI tool (DAX) reported a trend towards reducing burnout using the Oldenburg Burnout Inventory and a significant reduction in average time spent documenting notes. However, analysis was restricted to only include participants who completed the survey and used DAX greater than 60% of the time for evaluating burnout (28% or 23 participants) and additionally if there was data available on time in notes (22% or 19 participants).^12^ Another study evaluated the impact of DAX in a limited pilot of 12 dermatology physicians and physician assistants. While limited by a low survey response rate (60% or 6 clinicians) the authors concluded the majority of respondents were satisfied with documentation turnaround time, and stated the ambient AI tool “significantly improved” the overall quality of experience with patients.^13^ These studies support that ambient AI may be beneficial; however the results may not be directly relatable to our study in that they utilized a different ambient AI vendor with different product features and user experience, and were smaller studies limited to a specific medical specialty. In addition, Abridge contains several unique features compared to DAX that may have contributed to these striking findings, including: the ability to generate notes for multilingual visits where languages other than English were spoken (even without a translator), instant auditability where a clinician can highlight any AI-generated prose and immediately see the supporting evidence in the transcript and hear the audio, faster note turnaround times, and the ability to record and create multiple notes at the same time (eg, for an emergency room or primary care practice where multiple patients are being evaluated simultaneously).

One single-center cohort study that evaluated the impact of ambient AI platforms on a multispecialty clinician population utilized a mixed AI and human scribe solution (DAX) and showed mixed positive and negative effects of the technology.^14^ In that study, while there was an association with improved provider engagement, in work relative value units generated by providers, there was also and an increase in the after-hours time spent in the EHR in the intervention group, in contrast to our study which demonstrated decreased time spent in the EHR. Because the AI tool used in the former study required a human reviewer to edit the draft note prior to routing to the provider for final review, there may have been a long enough lag in note turn-a-round time resulting in providers potentially spending after-hours time to finalize notes. Abridge drafted notes in near real-time (median draft note generation time at our institution was 76 seconds in July 2023 and improved to 38 seconds by April 2024), likely allowing providers the ability to complete the note in a timelier fashion, with some completing notes even prior to the next patient visit.

While our study included a large number of participants, diversity in medical specialty, and over 50% survey response rates, there are some limitations. The surveys used for this study were originally designed for internal institutional business and operations audiences, rather than for formal research. As a result, pre-implementation survey responses were anonymous, and questions asked in the initial and follow-up surveys contained wordage that prohibited direct comparison of all but 2 survey items, which resulted in analysis to signal trends instead of associations for clinician satisfaction, cognitive load, burnout, and work outside of work. Given the anonymity of the pre-implementation survey, we were unable to account for the dependence in the groups between pre- and post-implementation survey data. This may have led us to underestimate the variability in the data to some degree. However, this should have a negligible effect on the estimated effect of ambient AI use on documentation experience, which showed a strong association. Ideally, the survey items should have undergone content validation prior to deployment. The pre- and post-implementation surveys also differed in the number and content of questions, which further limited our ability to compare responses pre- and post-intervention. Only 2 of the questions for each of the surveys were concordant enough to be compared with statistical analysis. Future iterations of similar research could benefit from standardized questions across surveys with validated measures for satisfaction, cognitive load, and burnout, use of EHR-based efficiency data, and a control group to better infer causality from the observed changes. In addition, the early adopter cohort of the AI ambient tool may have differed in clinical workload, comfort with technology, burnout, and documentation burden compared to non-participating clinicians. Results may not generalize to clinicians less interested in AI or healthcare information technology. Finally, as this study was a quality improvement study limited to a single academic medical center, the results may not be generalizable to other institutions or practice settings.

The present study focused on the subjective perceptions of clinicians on the use of ambient AI documentation platforms in clinical practice. Future research could report on more objective measures related to AI documentation technology, such as exploring time spent on documentation, time to note closure, note quality, number of patients seen, time spent in the EHR outside of work, and effects on revenue generation. Further studies could also address clinician readiness for adopting such technology, prior to implementation. This study also focused on short-term use of these tools (less than 1 year for all participants). Longer-term studies of this technology are needed to understand the degree to which there is sustained impact. Studies comparing impact across available and emerging ambient AI tools are needed and can help identify foundational product features needed to maximally support clinicians during their workday. Comparison of ambient AI tools with more traditional technology, such as voice recognition technology and in-person scribes may also be desirable. Furthermore, because this technology is evolving rapidly, often over weeks to months, in quality of note generation and new product features that for example customize clinical documentation to the specific needs of each specialty, research will need to be conducted at a more frequent cadence to properly evaluate impact on clinicians, patients and the greater healthcare ecosystem.

## Conclusion

Implementing the ambient AI documentation platform Abridge significantly improved clinicians’ perceptions of documentation efficiency, reduced after-hours work, and enhanced job satisfaction across various specialties within a short timeframe. While these findings are promising, they should be interpreted cautiously due to study limitations. Further research using validated measures and diverse settings is necessary to substantiate these results and explore the long-term impact of ambient AI on clinician workload and burnout.

## Supplementary Material

ooaf013_Supplementary_Data

## Data Availability

The data underlying this article will be shared on reasonable request to the corresponding author.
