# Supplementary material for: Enhancing clinical documentation with ambient artificial intelligence: a quality improvement survey assessing clinician perspectives on work burden, burnout, and job satisfaction
Source: JAMIA Open. 2025 Feb 21;8(1):ooaf013. doi: 10.1093/jamiaopen/ooaf013 (PMC11843214; doi:10.1093/jamiaopen/ooaf013)
Supplement: ooaf013_Supplementary_Data [file ooaf013_supplementary_data.zip › Abridge All Survey Response Distributions - Revision_1.docx]

**Distribution of Responses - Pre-Intervention Survey (N = 93)**

| Survey item | Strongly agree n (%) | Agree n (%) | Neither agree nor disagree n (%) | Disagree n (%) | Strongly disagree n (%) |
| --- | --- | --- | --- | --- | --- |
| I find my current documentation workflow easy to use for patient visits | 7 (7.5%) | 33 (35.5%) | 27 (29.0%) | 17 (18.3%) | 9 (9.7%) |
| I usually complete the note before the next patient visit | 8 (8.6%) | 5 (5.4%) | 11 (11.8%) | 32 (34.4%) | 37 (39.8%) |
| Documenting in O2 negatively impacts my patient care | 14 (15.1%) | 33 (35.5%) | 31 (33.3%) | 12 (12.9%) | 3 (3.2%) |
| I regularly spend time documenting outside of clinical hours (after hours/pajama time) because there's not enough time during clinical hours. | 49 (52.7%) | 28 (30.1%) | 4 (4.3%) | 9 (9.7%) | 3 (3.2%) |
| Documenting outside of clinical hours bothers me | 74 (79.6%) | 13 (14.0%) | 5 (5.4%) | 1 (1.1%) | 0 (0%) |
| Generally documentation causes me stress. | 36 (38.7%) | 42 (45.2%) | 11 (11.8%) | 4 (4.3%) | 0 (0%) |
| I am at risk for burnout due to documentation | 31 (33.3%) | 39 (41.9%) | 16 (17.2%) | 5 (5.4%) | 2 (2.2%) |

**Distribution of Responses - Post-Intervention Survey (N = 99)**

| Survey item | Strongly agree n (%) | Agree n (%) | Neither agree nor disagree n (%) | Disagree n (%) | Strongly disagree n (%) |
| --- | --- | --- | --- | --- | --- |
| Abridge has made my current documentation workflow easy to use for patient visits | 42 (42.4%) | 38 (38.4%) | 15 (15.2%) | 3 (3.0%) | 1 (1.0%) |
| With Abridge, I could complete the note before the next patient visit | 14 (14.1%) | 29 (29.3%) | 23 (23.2%) | 22 (22.2%) | 11 (11.1%) |
| Abridge has improved my patient care by decreasing the documentation burden | 34 (34.3%) | 42 (42.4%) | 16 (16.2%) | 5 (5.1%) | 2 (2.0%) |
| Abridge has decreased the time I spend documenting outside of clinical hours (i.e., after hours, non-clinic days) | 34 (34.3%) | 38 (38.4%) | 16 (16.2%) | 9 (9.1%) | 2 (2.0%) |
| Abridge has decreased the stress of documentation. | 44 (44.4%) | 35 (35.4%) | 14 (14.1%) | 5 (5.1%) | 1 (1.0%) |
| Abridge has decreased my risk for burnout due to documentation. | 30 (30.3%) | 36 (36.4%) | 20 (20.2%) | 12 (12.1%) | 1 (1.0%) |
| Abridge has improved the quality of my documentation. | 14 (14.1%) | 26 (26.3%) | 27 (27.3%) | 23 (23.2%) | 9 (9.1%) |
| Abridge has increased my satisfaction at work. | 24 (24.2%) | 39 (39.4%) | 30 (30.3%) | 6 (6.1%) | 0 (0%) |
| How likely are you to recommend Abridge to a friend or colleague? | 55 (55.6%) (Very likely) | 37 (37.4%) (Somewhat Likely) | 7 (7.1%) (Not Likely) |  |  |
| I feel that I could add at least one more patient encounter to my clinic session if urgently needed. | 21 (21.2%) | 26 (26.3%) | 21 (21.2%) | 21 (21.2%) | 10 (10.1%) |
| (If Strongly agree or Agree was answered on previous question): I estimate the number of patient encounters I could add to my clinic session is: | 0% (4+) | 14.9% (3) | 48.9% (2) | 36.2% (1) |  |
