# Supplementary material for: Enhancing clinical documentation with ambient artificial intelligence: a quality improvement survey assessing clinician perspectives on work burden, burnout, and job satisfaction
Source: JAMIA Open. 2025 Feb 21;8(1):ooaf013. doi: 10.1093/jamiaopen/ooaf013 (PMC11843214; doi:10.1093/jamiaopen/ooaf013)
Supplement: ooaf013_Supplementary_Data [file ooaf013_supplementary_data.zip › Abridge Post Survey.pdf]

Abridge Post-Survey

A A A

Please complete the survey below.

Thank you!

Please provide your name.

* must provide value

What is your current documentation workflow in O2? *(Please select all that apply.)*

* must provide value

- [ ] Student Documentation
- [ ] Resident Documentation
- [ ] Manual Typing
- [ ] Templates & Dotphrases
- [ ] Dragon Dictation to Deskop
- [ ] Dragon Dictation to Haiku/Canto
- [ ] In-Person Scribe
- [ ] DAX
- [ ] Abridge
- [ ] Other

Abridge has made my current documentation workflow easy to use for patient visits. *(Please select one.)*

* must provide value

- ○ Strongly Agree
- ○ Agree
- ○ Neither Agree nor Disagree
- ○ Disagree
- ○ Strongly Disagree
- ○ Not Relevant to My Experience

**With Abridge, I could complete the note before the next patient visit. *(Please select one.)***<br>* must provide value	◯ Strongly Agree<br>◯ Agree<br>◯ Neither Agree nor Disagree<br>◯ Disagree<br>◯ Strongly Disagree<br>◯ Not Relevant to My Experience
**Abridge has improved my patient care by decreasing the documentation burden. *(Please select one.)***<br>* must provide value	◯ Strongly Agree<br>◯ Agree<br>◯ Neither Agree nor Disagree<br>◯ Disagree<br>◯ Strongly Disagree<br>◯ Not Relevant to My Experience
**Abridge has decreased the time I spend documenting outside of clinical hours (ie, after hours, non-clinic days). *(Please select one.)***<br>* must provide value	◯ Strongly Agree<br>◯ Agree<br>◯ Neither Agree nor Disagree<br>◯ Disagree<br>◯ Strongly Disagree<br>◯ Not Relevant to My Experience
**Abridge has decreased the stress of documentation. *(Please select one.)***<br>* must provide value	◯ Strongly Agree<br>◯ Agree<br>◯ Neither Agree nor Disagree<br>◯ Disagree

- Strongly Disagree
- Not Relevant to My Experience

Abridge has decreased my risk for burnout due to documentation. *(Please select one.)*

* must provide value

- Strongly Agree
- Agree
- Neither Agree nor Disagree
- Disagree
- Strongly Disagree
- Not Relevant to My Experience

Abridge has improved the quality of my documentation. *(Please select one.)*

* must provide value

- Strongly Agree
- Agree
- Neither Agree nor Disagree
- Disagree
- Strongly Disagree
- Not Relevant to My Experience

Abridge has increased my satisfaction at work. *(Please select one.)*

* must provide value

- Strongly Agree
- Agree
- Neither Agree nor Disagree
- Disagree
- Strongly Disagree
- Not Relevant to My Experience

How likely are you to recommend Abridge to a friend or colleague? *(Please select one.)*

* must provide value

- ◯ Not Likely
- ◯ Somewhat Likely
- ◯ Very Likely

I feel that I could add at least one more patient encounter to my clinic session if urgently needed. *(Please select one.)*

* must provide value

- ◯ Strongly Agree
- ◯ Agree
- ◯ Neither Agree nor Disagree
- ◯ Disagree
- ◯ Strongly Disagree
- ◯ Not Relevant to My Experience

Please share any additional comments on how Abridge has impacted you:

Please share any suggestions or idea for how to make Abridge even more valuable to you and/or your colleagues.

Submit
