# Supplementary material for: Enhancing clinical documentation with ambient artificial intelligence: a quality improvement survey assessing clinician perspectives on work burden, burnout, and job satisfaction
Source: JAMIA Open. 2025 Feb 21;8(1):ooaf013. doi: 10.1093/jamiaopen/ooaf013 (PMC11843214; doi:10.1093/jamiaopen/ooaf013)
Supplement: ooaf013_Supplementary_Data [file ooaf013_supplementary_data.zip › Abridge Pre Survey.pdf]

A A A

THE UNIVERSITY OF KANSAS HEALTH SYSTEM

Abridge Pre-Survey

Please complete this brief survey about your current documentation experience. It should take less than 5 minutes to complete.

Thank you!

What is your current documentation workflow in O2?

(Please select all that apply.)

* must provide value

- ☐ Student documentation
- ☐ Resident documentation
- ☐ Manual typing
- ☐ Templates & dotphrases
- ☐ Dragon dictation to desktop
- ☐ Dragon dictation to Haiku/Canto
- ☐ In-person scribe
- ☐ AI & virtual scribe ambient documentation
- ☐ AI only ambient documentation
- ☐ Other

I find my current documentation workflow easy to use for patient visits.

(Please select the best response.)

- ○ Strongly agree
- ○ Agree
- ○ Neither agree nor disagree

* must provide value

- ◯ Disagree
- ◯ Strongly disagree
- ◯ Not relevant to my experience

I usually complete the note before the next patient visit.
(Please select the best response.)

* must provide value

- ◯ Strongly agree
- ◯ Agree
- ◯ Neither agree nor disagree
- ◯ Disagree
- ◯ Strongly disagree
- ◯ Not relevant to my experience

Documenting in O2 negatively impacts my patient care.
(Please select the best response.)

* must provide value

- ◯ Strongly agree
- ◯ Agree
- ◯ Neither agree nor disagree
- ◯ Disagree
- ◯ Strongly disagree
- ◯ Not relevant to my experience

I regularly spend time documenting outside of clinical hours (after hours/pajama time) because there's not enough time during clinical hours.
(Please select the best response.)

* must provide value

- ◯ Strongly agree
- ◯ Agree
- ◯ Neither agree nor disagree
- ◯ Disagree
- ◯ Strongly disagree
- ◯ Not relevant to my experience

Documenting outside of clinical hours bothers me.
(Please select the best response.)

- ◯ Strongly agree

* must provide value

- Agree
- Neither agree nor disagree
- Disagree
- Strongly disagree
- Not relevant to my experience

Generally, documentation causes me stress. (Please select the best response.)

* must provide value

- Strongly agree
- Agree
- Neither agree nor disagree
- Disagree
- Strongly disagree
- Not relevant to my experience

I am at risk for burnout due to documentation.

(Please select the best response.)

* must provide value

- Strongly agree
- Agree
- Neither agree nor disagree
- Disagree
- Strongly disagree
- Not relevant to my experience

Is there anything else about your documentation experience you'd like to share at this time?

Submit

Powered by REDCap
